# Colonisation of the Non-Indigenous Pacific Oyster *Crassostrea gigas* Determined by Predation, Size and Initial Settlement Densities

**DOI:** 10.1371/journal.pone.0090621

**Published:** 2014-03-24

**Authors:** Luke H. Hedge, Emma L. Johnston

**Affiliations:** Sydney Institute of Marine Science and the Evolution and Ecology Research Centre, School of Biological Earth and Environmental Science, The University of New South Wales, Sydney, Australia; Dauphin Island Sea Lab; University of South Alabama, United States of America

## Abstract

Survival of incipient non-indigenous populations is dramatically altered by early predation on new colonisers. These effects can be influenced by morphological traits, such as coloniser size and density. The Australian non-native Pacific Oyster *Crassostrea gigas* is generally more fecund and faster growing compared to the native *Saccostrea glomerata* found in the same habitat. It is therefore important to quantify how the two species differ in survival across coloniser density and predation gradients. This information could become pertinent to the management of wild and aquaculture populations of the non-native *C. gigas*. Using a field-based factorial experiment we model the survival of incipient populations of both the native *S. glomerata* and the non-indigenous *C. gigas* as a function of coloniser density, predator reduction and individual size. Unexpectedly, survival of the non-indigenous *C. gigas* increased compared to *S. glomerata* when individuals were larger. The proportional survival of newly colonised oyster populations also increased with larger initial populations, regardless of species identity. Further, predator reduction resulted in increased survival of both oyster species, irrespective of coloniser size or initial density. Here we quantitatively demonstrate the effects of recruit density and size on enhancing the survivability of incipient oyster populations.

## Introduction

Numerical modeling, and several recent investigations into grassland and forest biological invasions, highlights the need to compare ‘invasibility’ between sites and systems in the context of coloniser densities resulting from variable propagule supply [1]. Temperate and tropical reef communities are analogous to terrestrial plant systems [2], yet there are few examples of marine ‘non-indigenous seed addition’ type experiments that are common in grasses and other plant systems [3]. As in terrestrial systems, human activities act to spread non-indigenous species (NIS), mainly through shipping and aquaculture, leading to variation in coloniser density [4]. Theoretical modeling and lab mesocosm experiments are now drawing links between variation in colonisation density, NIS establishment/survival and spread [5], [6], [7], [8], [9], [10].

Colonisation processes need to be understood within the context of the surrounding biotic and abiotic environment [11], [12], [13]. Herbivory in plant systems has been shown to interact with coloniser abundance to determine the establishment success of a variety of taxa [11]. Predation can also directly regulate incipient NIS populations [14], or act indirectly to enhance community stability and invasion resistance [15]. Predation pressure therefore warrants further consideration if we are to fully understand the complex relationship between colonisation density and invasion.

The NIS bivalve *Crassostrea gigas* has been introduced, deliberately or by accident, to 79 countries around the world [16]. Twenty-four of these countries now have self-sustaining populations. In south-east Australia, small populations of *C. gigas*, are generally found interspersed between the Australian native Sydney rock oyster *Saccostrea glomerata* on natural rocky reefs and other populations can be found on commercial aquaculture leases [17]. Interactions between propagule pressure and predation may explain this patchy distribution [16], [18]. Evidence suggests the fecundity of *C. gigas* is approximately 80 % greater than the similar Australian native *S. glomerata* [19]. The greater larval abundance and predicted increase in recruitment of the non-indigenous *C. gigas* may enhance early population survival through the avoidance of inverse density dependant processes and deleterious environmental stochasticity [2], [20]. While it is logistically difficult to manipulate the abundance of larvae entering a marine system (but see [2], [21], [22]), we can usually manipulate the abundance of early colonisers that have recently established. The abundance of these colonisers predictably increases with greater propagule pressure in various marine invertebrate taxa, and so we use ‘coloniser abundance’ or ‘colonisation’ here as a simple proxy for propagule pressure. Increased colonisation may be predicted to create a more complex oyster reef, reducing predator efficiency [23], [24], [25], and increasing food delivery through a more complex boundary layer [26]. Higher colonisation rates may then directly increase population survival by i) increasing the probability of at least some new recruits surviving stochastic biotic and abiotic processes ii) decreasing predator performance over an increasingly complex habitat or iii) increasing food flux to underlying oysters in a more dense, complex reef structure. Alternatively, higher coloniser abundance may in fact increase the proportional mortality of individuals through a type III functional response [27], making predictions regarding predator related oyster mortality less certain. Note that these effects, whatever they may be, would be predicted to be similar between both the native and NIS oysters.

Effects of increased coloniser density may also be stronger with larger coloniser size. This is particularly pertinent, as *C. gigas* has been shown to grow over 60% faster than *S. glomerrata* [28]. While *r*-selected traits like faster growth, have been implicated as conferring greater ‘invasibility’ [29], trade-offs exist that make it hard to predict NIS proliferation based on these traits alone [30]. *C. gigas*, for example, has been found to be a poor competitor under harsher environmental conditions such as long emersion times [19], while other bi-bivalve species with *r*-type life history traits also have poor resistance to predatory pressure [30]. There is some evidence to suggest that the shell of *C. gigas* is thinner than that of *S. glomerata* particularly in larger (> 80 mm) oysters [31], and may therefore be more susceptible to predation, much like the well-studied *Crassostrea ariakensis/Crassostrea virginica* system in the Chesepeake Bay, USA [30]. So while *C. gigas* can outcompete *S. glomerata* by growing faster under favorable conditions [19], the invasion potential of this species may vary with predatory pressure and recruit density. It then becomes important to test the size specific responses of the non-indigenous *C. gigas* to predation and coloniser density, and compare these effects to the native *S. glomerata* in a factorial, field based experiment.

To test for the species specific, independent and interactive effects of coloniser density, predation and size on the survivorship of the non-indigenous *C. gigas* and the morphologically similar *S. glomerata*, we conducted a factorial field experiment in which we manipulated all four factors on a pre-established oyster reef. We estimated population survival after 12 weeks (or ‘invasion success’ for *C. gigas*) by measuring the proportional survival and total abundances of both *C. gigas* and *S. glomerata* and explored the predictions that i) proportional survival and total abundances of oysters increase with greater recruit density ii) larger oysters of both species show greater survival and iii) effects of size, recruitment density and predation are similar for both *C. gigas* and *S. glomerata*.

## Methods

### Ethics Statement

This study was conducted within Chowder Bay, a small inlet on the northern side of Sydney Harbour, NSW, Australia (33°50’19.80”S, 151°15’16.50”E). The owner of the land (Sydney Harbour Foreshore Trust) gave permission to conduct the study on this site. No specific permissions were required for these locations/activities, and studies did not involve endangered or protected species.

### Study System

The proximity of Chowder Bay to the harbour entrance ensured good tidal flushing and relatively clean seawater. A 50 m stretch of the shoreline, consisting mostly of small (< 10 kg) boulders, on the eastern side of Chowder Bay was used for all manipulations. The sessile invertebrate community within the boulder field was dominated by *S. glomerata* reef, but also contained foliose and encrusting algae (predominantly the Chlorophyte *Ulva lactuca*), bryozoans (e.g. *Watersipora subtorquata*), solitary ascidians (including *Pyura preaputialis*), grapsid crabs and barnacles (*Balanus variegates* and *Balanus trigonus*, *authors unpub.*). The ranges of *S. glomerata* and *C. gigas* do overlap on the south east coast of Australia.

### Density and predation manipulation

This study factorially manipulated oyster size, density, species identity, and predator access using transplanted oysters placed into small plots located on the boulders within the Chowder Bay rocky boulder field. Manipulations of juvenile *C. gigas* and *S. glomerata* population densities were conducted from August-September 2010. Juvenile, diploid, *C. gigas* and *S. glomerata* of 3–5 mm and 12–15 mm length were obtained commercially. The stock were spawned and raised in similar conditions and were grown in the same locations within the Port Stephens estuary on the south east coast of Australia. The 3–5 mm size class represented a newly colonized oyster of approximately 1–1.5 months age, while the 12–15 mm class represented the average size of *C. gigas* after 1.5–2 months or *S. glomerata* after 2–2.5 months [28]. *C. gigas* and *S. glomerata* of both size classes were glued to boulders in three increasing densities (5, 20, 30 juveniles) within a 10 cm^2^ plot using marine epoxy glue (International Epiglue). Similar studies show no effects of gluing bivalves to hard substrate when the bivalves ability to gape is unhindered [14]. Oysters were glued directly onto rock or adult oysters depending on the random positioning. If the oyster was randomly positioned over algae or colonial animals, these pre-established animals and algae were removed prior to gluing. Each boulder was approximately 30×30×30 cm, weighed approximately 5 kg and was observed not to move under heavy swell. Each replicate patch was randomly assigned to a cage, no cage or a cage control treatment in a fully orthogonal design, with seven replicates per size/species/cage/density combination; 252 boulders were therefore manipulated and returned to the reef. Cages were constructed from 1 cm diameter plastic mesh, 11 cm^2^ at the base, and 8 cm high. In order to deter benthic gastropod predators, a commercial non-toxic resin (Tanglefoot) was applied around the base of the cages [32]. Cage controls consisted of a cage roof and one side only, with resin applied along one randomly chosen edge. Due to logistical constraints of working within a small boulder field, a separate control specifically for the application of resin was not conducted and we define our ‘cage control’ as testing for the combined effect of caging and the application of resin. Cages and cage controls were nailed into the rock so that they covered the entire patch. Un-manipulated treatments were marked with a nail hammered directly into the rock. Cages were cleaned weekly, and larger invertebrate snails that found their way into the cages were removed. Importantly, cages were a form of predator reduction, not exclusion. Smaller intertidal invertebrates and juvenile fish could possibly have gained access, although the common oyster predators of south east Australia, including adult Bream *Acanthopagrus australis*, Snapper *Chrysophrys auratus*, Toadfish *Tetractenos* spp. and Leatherjackets *Monocanthus chinensis*, would have been completely excluded [33].

Boulders were haphazardly replaced onto the low intertidal zone of the boulder reef with the experimental patch approximately horizontal. The manipulated boulder field occupied a 3 m wide and 40 m long area of the reef in the low intertidal, running parallel to the shoreline. Due to slope of the shoreline and the tidal period in southern Australia, differences in emersion times between experimental populations at the bottom of the experimental area and those at the top were negligible (approximately 20 min). All oysters were removed and destroyed at the conclusion of the experiment. The addition of non-indigenous species into new areas presents a serious ethical concern and animals had to be removed prior to reaching sexual maturity (usually occurring only after 12–20 months, *authors unpublished*). Therefore this experiment did not attempt to test long-term survival of these populations. It is important to note that Pacific Oysters are present in Sydney Harbour, with patchy distributions found throughout the estuary.

### Census and statistical analysis

In the context of this study we treat population survival or ‘invasion success’ as a probabilistic concept, rather than a binary certainty (i.e. a system is invaded or is not). Most of our experimental populations had at least one oyster surviving to 12 weeks, however the probability of one oyster surviving to maturity and reproducing would be far lower than that of 30 oysters. For this reason, we argue that quantifying proportional survival of oysters in our experimental populations is the most appropriate measure of population survival, rather than simple presence/absence measurements.

Surviving oysters were counted after 12 weeks. Storm damage reduced the number of replicates per treatment combination to between 3–6 and created an unbalanced design (Table 1).

**Table 1:** Storm activity and human tampering reduced the number of replicates in this experiment.

Treatment	Level	Replicate patches
**Species**	*C. gigas*	93
	*S. glomerata*	88
**Size**	Large	90
	Small	91
**Cage**	Caged	53
	Cage control	65
	Un-caged	53
**Density**	5	62
	20	73
	30	46

Numbers of plots remaining for each treatment combination are presented below.

#### Model formulation

Oyster proportional survival was analysed using Generalized Linear Modeling (GLM) and Maximum Likelihood techniques. The survival of oysters at 12 weeks within each patch was modeled as a function of species (two levels; *S. glomerata* and *C. gigas*), size (two levels: small and large), density (three levels; 5, 20, 30), and predator reduction (three levels; cage, cage control and uncaged). The model was constructed using a binomial function with a logit link [34], [35]. Model reduction was used to remove terms that contributed little to model fit [34], leaving an optimal model from which to interpret effects following the methods of Zuur et al. [36]. R Code for our model selection technique is presented in Supplemental Materials to this paper (Code S1). Graphical representation of our model predictions are also found in Figure S1.

## Results

Generally, the glued valve of oysters remained within the plots, indicating the oysters had died and not simply washed away from the glue. We did not observe any forensic evidence of predation (drilled shells, shell fragments) in our plots, however, over the time period of our experiment these fragments would have been degraded and removed.

The optimal model for predicting oyster survival consisted of all the low order terms (Density, Cage, Species, Size) as well as several high order interactions between Density, Size and Species (Table 2). Higher order three and four way interactions were found not to contribute to model fit and were excluded during model optimization. Graphical model predictions are presented in Figure S1 to this paper, and simple means and standard errors are presented here for simplicity.

**Table 2:** Analysis of Deviance, testing the contribution of each component to the overall model.

	LR χ2	df	p(> χ2 )
Density	18.16	2	<0.001
Cage	119	2	<0.001
Species	11.097	1	<0.001
Size	2.45	1	0.12
Dens. × Size	17.945	2	<0.001
Sp. × Size	6.72	1	<0.01
null deviance = 1880.9181*d f*			
resid. deviance = 1084.8172*d f*			
θ = 5.6			

Presented is the optimal model only. Terms found not to contribute are not included.

**Figure 1:**
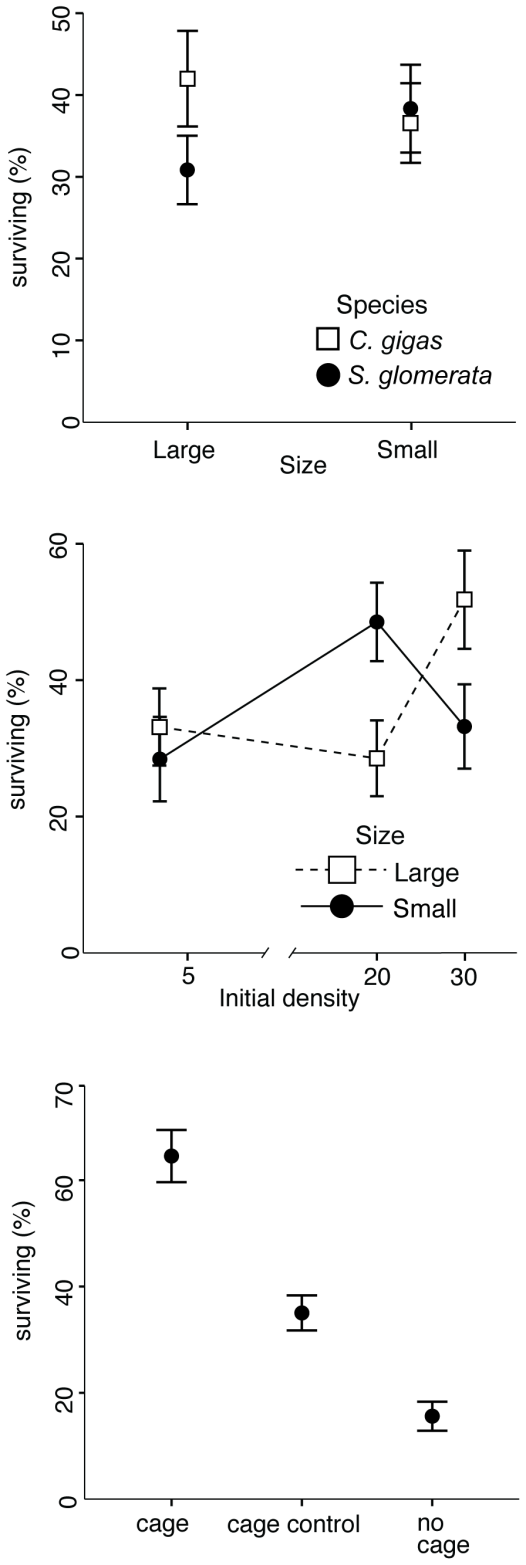
Proportional survival of transplanted oysters as a function of density, species, size and caging. Replicate numbers are presented in Table 1. Error bars represent two S.E around the mean.

The species of transplanted oysters could predict survival probabilities in conjunction with the size of the transplanted individual (Table 2, 3). The probability of a large *C. gigas* surviving until census was 45 %, almost 20 percentage points higher than a large *S. glomerata* (25 %, Table 3, Fig 1). There were no differences in predicted survival rates between *C. gigas* and *S. glomerata* when oyster size was small (Table 2, Fig 1). Forty two percent of larger *C. gigas* remained in our plots at the time of census, whilst only 31% of larger *S. glomerata* remained (Fig 1).

**Table 3:** Estimates from binomial General Lin- ear Modeling.

	Estimate ±(SE)
Intercept	–1.32 (0.50)**
Density: 20‡	–0.36 (0.50)
Density: 30‡	0.90(0.50)†
Cage: caged§	2.63(0.27)***
Caged: ca. cont.§	0.82(0.24)***
Species: S.R.O.	−0..93(0.28)*
Size: Sm.	−1.08(0.69)†
Dens.: 20×Size: Sm.	1.56(0.73)*
Dens.: 30×Size: Sm.	−0.19(0.72)
Species: S.R.O.×Size: Sm.	1.03(0.40)*
N	181

Parameter estimates are given on the scale of the model (logits). Exponentiating these terms will convert these estimates to log- odds.

† significant at *p*<.10; * *p*<.05; ** *p*<.01; *** *p*<.001.

‡ the reference category for the Density treatment is five oysters.

§ the reference category for the Caging treatment is un-caged.

Coloniser density also predicted survival of oysters, however this varied not between species, but between size classes (Table 2, 3, Fig. 1). The probability of survival increased dramatically when larger oysters were transplanted at higher densities (Table 2, 3, Fig 1). A large oyster was predicted to survive until census 32, 25 and 54 % of the time when 5 (low density), 20 (mid density) or 30 (high density) oysters were transplanted into the plots (Table 3, Fig 1). Conversely, survival of smaller oysters did increase initially, however survival seemed to decrease within the higher density plots (Fig 1). An individual small oyster had a 21, 46 and 36 % chance of surviving in each of plots of increasing density (Table 3, Fig 1).

Caging had a predictable effect on oyster survival. Fifteen percent of oysters in the uncaged treatments remained at the end of the study, whilst almost 65 % remained in caged plots (Fig 1). Predicted individual survival aligned closely with our observed data and oysters in uncaged plots had a 15.4 % chance of surviving, whilst in caged plots the individual survival rate was 64.5 % (Table 3). Oysters in our half caged, control plots had a 35 % chance of mortality, which was different to the uncaged plots at the p < 0.05 level (Table 3, Fig 1). This indicated that cages did have an effect on survival that seemed to be independent of predator reduction (however see *Discussion*).

## Discussion

The most striking result from this study is the greater survival of large NIS *C. gigas*. This was not predicted, but research on the *C. gigas/S. glomerata* system on southern Australian shores is lacking (but see [19 17]). Predation did not determine this pattern, and so we are confident that predator behavioral responses to the different oyster species are unlikely. In the current study we experimentally manipulated densities of oyster settlers to those found in areas of high abundance in natural systems (approximately 3000 recruits.m^2^). In recent invasions by *C. gigas* into the Frisian Wadden Sea for example, densities of *C. gigas* settler abundance ranged from 1.5–1460 oysters.m^2^ onto established mussel reefs (measured yearly [37]), while on the French coast, settler abundance ranged from 4–4550 oysters.m^2^ [38].

Krassoi et al. [19] suggests a trade-off exists that allows *C. gigas* to outcompete the native *S. glomerata*, however only in the low intertidal where oysters are not subjected to the same abiotic stress found in higher intertidal areas. In these harsher conditions *C. gigas* suffers increased mortality [19]. In the current study, increasing the size of *C. gigas*, increased survival. We would predict that increasing size would increase the resilience of the species to abiotic stress. It may also increase shading and the retention of water throughout the plots during low tides. Survival of *S. glomerata* did not change with size (Fig. 1) and this species is generally thought not to be affected as much by desiccation stress, compared with the non-indigenous *C. gigas.*

Seminal work by Grabowski [39], Grabowski et al. [40], Grabowski and Powers [24] and Hughes and Grabowski [41] highlight the role of habitat complexity in mediating predation, survival and trophic transfer on reefs of other oyster species. In the current study, effects of size and species were observed regardless of caging. We also observed general increases in survivorship with increasing density, albeit these patterns differed between size classes. Much like Ruesink [18], however, we also observed smaller whelk and crab predators within several cages during this experiment, indicating that cages were more of a predator reduction treatment. It is these smaller whelks and crabs that may have contributed to the different survival probabilities between larger *C. gigas* and *S. glomerata.* Our data and modeling may therefore support Ruesink’s [18] proposition that increasing the complexity of underlying oyster reef habitat (in Ruesink’s [18] case through the presence of nearby neighbors, not increases in size) may have increased survival of *C. gigas* by increasing resilience to abiotic stress and through reductions in predator efficiency. If this were the case, then our results would then suggest the effects size and complexity are not conserved between the NIS and native oyster species. This would be a novel research direction for the *C. gigas/S. glomerata* system on south-east Australian shores.

It is also important to note that increasing the coloniser density might have very different consequences for predators depending on whether these occur across small plots, such as those used in this study, or occur at a whole-shore scale. At a whole-shore scale, prey at high density may actually saturate predators. In the small plots used in this experiment, predators could be attracted to plots at high density. This could make the interpretation of our lack of density by predation interaction less generalizable at larger spatial scales, and is something to investigate in the future. It is also important to note the effect of our ‘half cage’ controls, which increased predicted survival of oysters over that of our un-manipulated plots. In the current study, cages were a form of predator reduction. It could be predicted that our cage controls acted as an intermediary treatment between fully caged and completely exposed, creating a gradient of predator access. While this calls into question the efficacy of simple ‘half cage controls’, this model of predator reduction does fit with our statistical evidence where survival increased in a stepwise fashion from fully exposed to fully caged.

The pathogen load associated with the native *S. glomerata* cannot be ignored when predicting drivers of survival differences. While there are very few known diseases of *C. gigas* in south east Australia, a variety of pathogens afflict *S. glomerata*, including QX disease (a paramyxean protozoan, *Marteilia sydneyi*), Winter Mortality (a haplospridian protozoan, *Bonamia roughleyi*), and mud worm infestation (*Polydora websteri*) [42]. These parasites do not affect the non-indigenous *C. gigas*. Winter mortality in particular has been shown to cause dramatic increases in *S. glomerata* mortality in the low intertidal [43], the tidal level at which the current study was located. If oyster survival is mediated by pathogen abundance, the non-indigenous *C. gigas* may escape mortality in much the same way as the paradigmatic ‘Enemy Release hypothesis’ that has received much critical debate [44]. This model is supported by evidence from Northwest Europe, where the non-indigenous *C. gigas* populations have generally less parasite related mortality than European natives. This has been implicated as one factor mediating the prolific *C. gigas* spread throughout the area [23]. A similar model may operate in the *C. gigas*/*S. glomerata* system in southern Australia, however it is important to note the spread of the Pacific Oyster Herpes Virus that currently infects several estuaries in South East Australia. If this virus continues to spread and affect only Pacific Oyster populations, then apparent ‘enemy release’ processes may cease and populations of *C. gigas* may decline.

Increased substrate complexity created from increasing spat densities may also create a turbulent flow in which food particles become entrained [45]. An optimal density may exist for very early oyster recruits or naturally smaller recruits, such as that observed at intermediate densities of the small oysters in our current study. In this case, an intermediate density in which turbulent flow increases food flux to the benthos is beneficial to small oysters, but any subsequent increases in density outweigh this benefit through intra-specific competition. While this was not explicitly explored in the current study, density dependent intra-specific competition through reductions in food availability has been demonstrated previously in *C. gigas* [46], as well as a suite of other aquaculturally important bivalve species [47], [48]. Other forms of interference competition, such as bulldozing or smothering by conspecifics would have been negligible over the 12-week duration of the experiment. Under this model, however, larger oysters in high-density treatments would then also be predicted to be experiencing intra-specific competition for food. Greater energy reserves and enhanced shell protection in larger oysters may have acted to reduce direct mortality, however again this is speculative and the growth and physiology of this species at different ages and how this changes in competition with *S. glomerata* may be a novel research direction.

## Conclusions

Initial population establishment and survival is a key demographic property of all non-indigenous species. If an incipient non-indigenous population is able to survive through early establishment, when abiotic and biotic stress is greatest, then the probability of spread obviously increases dramatically. It is therefore important to understand how recruit density can alter the proportional survival of non-indigenous species. Both the non-indigenous *C. gigas* and the native *S. glomerata* had greater proportional survival rates, and abundances of surviving oysters were greatly increased in high density treatments at the conclusion of the experiment. Surprisingly, we also demonstrated that large *C. gigas* recruits had a greater survival rate when compared with the morphologically similar native *S. glomerata*, averaged across all other factors. Additionally, predator related mortality acted independently to drastically reduce the survival of both species regardless of density and size.

## Supporting Information

Figure S1 Effects under investigation were calculated and plotted absorbing the lower-order terms marginal to the term in question, and averaging over other terms in the model, using the effects package (Fox and Hong, 2003).
(DOCX)

Code S1 Model Selection procedures followed the methods of Zuur et al. (2009). Analysis of Deviance was used to test for the optimal model from which to interpret effects.
(DOCX)
