# Supplementary material for: Colonisation of the Non-Indigenous Pacific Oyster Crassostrea gigas Determined by Predation, Size and Initial Settlement Densities
Source: PLoS One. 2014 Mar 24;9(3):e90621. doi: 10.1371/journal.pone.0090621 (PMC3963846; doi:10.1371/journal.pone.0090621)
Supplement: Figure S1 — Effects under investigation were calculated and plotted absorbing the lower-order terms marginal to the term in question, and averaging over other terms in the model, using the effects package (Fox and Hong, 2003). (DOCX) [file pone.0090621.s001.docx]

Figure S.1. Effects under investigation were calculated and plotted absorbing the lower-order terms marginal to the term in question, and averaging over other terms in the model, using the effects package (Fox and Hong, 2003).
