# Supplementary material for: Colonisation of the Non-Indigenous Pacific Oyster Crassostrea gigas Determined by Predation, Size and Initial Settlement Densities
Source: PLoS One. 2014 Mar 24;9(3):e90621. doi: 10.1371/journal.pone.0090621 (PMC3963846; doi:10.1371/journal.pone.0090621)
Supplement: Code S1 — Model Selection procedures followed the methods of Zuur et al. (2009). Analysis of Deviance was used to test for the optimal model from which to interpret effects. (DOCX) [file pone.0090621.s002.docx]

**R code for Model Selection**

####Hedge and Johnston (2013) PLOS ONE

###SUPPLEMENTARY MATERIALS

#LIBRARIES

library(car)

library(effects)

#Model construction and optimization

mod.a<-glm(cbind(alive,munched)~Density+Cage+Species+Size+Density:Cage+Density:Species+Cage:Species+

Density:Size +

Cage:Size +

Species:Size +

Density:Cage:Species +

Density:Cage:Size +

Density:Species:Size +

Cage:Species:Size +

Density:Cage:Species:Size ,

data=data,family =quasibinomial)

> drop1(mod.a, test='Chisq')

Df Deviance scaled dev. Pr(>Chi)

<none> 949.59

Density:Cage:Species:Size 4 954.30 0.8184 0.936

>mod.b<-update(mod.a,.~.-Density:Cage:Species:Size)

>drop1(mod.b,test='Chisq')

Df Deviance scaled dev. Pr(>Chi)

<none> 954.30

Density:Cage:Species 4 970.46 2.8358 0.5857

Density:Cage:Size 4 986.15 5.5899 0.2319

Density:Species:Size 2 957.75 0.6056 0.7388

Cage:Species:Size 2 962.80 1.4912 0.4745

>mod.c<-update(mod.b,.~.-Density:Species:Size)

>drop1(mod.c,test='Chisq')

Df Deviance scaled dev. Pr(>Chi)

<none> 957.75

Density:Cage:Species 4 972.94 2.7081 0.6078

Density:Cage:Size 4 989.31 5.6276 0.2287

Cage:Species:Size 2 966.51 1.5622 0.4579

>mod.d<-update(mod.c,.~.-Density:Cage:Species)

>drop1(mod.d,test='Chisq')

Df Deviance scaled dev. Pr(>Chi)

<none> 972.94

Density:Species 2 978.93 1.0965 0.5780

Density:Cage:Size 4 1002.60 5.4316 0.2458

Cage:Species:Size 2 977.75 0.8814 0.6436

>mod.e<-update(mod.d,.~.-Cage:Species:Size)

>drop1(mod.e,test='Chisq')

Df Deviance scaled dev. Pr(>Chi)

<none> 977.75

Density:Species 2 983.46 1.0562 0.589713

Cage:Species 2 1013.66 6.6405 0.036143 *

Species:Size 1 1023.22 8.4089 0.003734 **

Density:Cage:Size 4 1007.48 5.4982 0.239891

>mod.f<-update(mod.e,.~.-Density:Species)

>drop1(mod.f,test='Chisq')

Df Deviance scaled dev. Pr(>Chi)

<none> 983.46

Cage:Species 2 1018.56 6.5451 0.03791 *

Species:Size 1 1028.05 8.3157 0.00393 **

Density:Cage:Size 4 1011.82 5.2890 0.25891

>mod.g<-update(mod.f,.~.-Density:Cage:Size)

>drop1(mod.g,test='Chisq')

Df Deviance scaled dev. Pr(>Chi)

<none> 1011.8

Density:Cage 4 1054.4 7.7931 0.099458 .

Cage:Species 2 1039.9 5.1399 0.076539 .

Density:Size 2 1117.3 19.2981 6.449e-05 ***

Cage:Size 2 1015.7 0.7158 0.699147

Species:Size 1 1053.3 7.5902 0.005869 **

>mod.h<-update(mod.g,.~.-Cage:Size)

>drop1(mod.h,test='Chisq')

Df Deviance scaled dev. Pr(>Chi)

<none> 1015.7

Density:Cage 4 1058.7 7.8762 0.096218 .

Cage:Species 2 1042.8 4.9652 0.083525 .

Density:Size 2 1122.7 19.5979 5.551e-05 ***

Species:Size 1 1056.8 7.5305 0.006066 **

>mod.i<-update(mod.h,.~.-Density:Cage)

>drop1(mod.i,test='Chisq')

Df Deviance scaled dev. Pr(>Chi)

<none> 1058.7

Cage:Species 2 1084.8 4.7045 0.095156 .

Density:Size 2 1162.4 18.7142 8.635e-05 ***

Species:Size 1 1096.8 6.8676 0.008777 **

>mod.j<-update(mod.i,.~.-Cage:Species)

>drop1(mod.j,test='Chisq')

Df Deviance scaled dev. Pr(>Chi)

<none> 1084.8

Cage 2 1754.8 118.989 < 2.2e-16 ***

Density:Size 2 1185.8 17.945 0.0001268 ***

Species:Size 1 1122.6 6.722 0.0095231 **

>Anova(mod.j,test='Chisq', type=3)

>summary(mod.j)

### PREDICTIONS WITH EFFECTS PACKAGE

>effect("Species:Size", mod.j)

Species*Size effect

Size

Species l s

p 0.4557201 0.3317030

s 0.2483267 0.3539714

>effect("Density:Size", mod.j)

Density*Size effect

Size

Density l s

5 0.3281415 0.2148135

20 0.2546679 0.4656857

30 0.5457983 0.3573454

>effect("Cage", mod.j)

t c p

0.1541965 0.7165775 0.2934407

**References**

Fox, J., Hong, J (2009). Effect Displays in R for Multinomial and Proportional-Odds Logit Models: Extensions to the effects Package. Journal of Statistical Software, 32(1), 1-24.
